# Assessing disease phenotypes in Behçet’s syndrome: insights from a multiple correspondence analysis

**DOI:** 10.3389/fimmu.2025.1605714

**Published:** 2025-06-20

**Authors:** Rosaria Talarico, Federica Di Cianni, Antonello Sulis, Diana Marinello, Valentina Lorenzoni, Marta Mosca

**Affiliations:** ^1^ Rheumatology Unit, Azienda Ospedaliero-Universitaria Pisana, Pisa, Italy; ^2^ Department of Medical Biotechnologies, University of Siena, Siena, Italy; ^3^ Institute of Management, Scuola Superiore Sant’Anna, Pisa, Italy

**Keywords:** Behcet syndrome, multi correspondence analysis (MCA), disease phenotypes, historical cohort study, clinical profiles

## Abstract

**Introduction:**

Behçet’s syndrome (BS) is a rare systemic vasculitis. Clinical manifestations in BS are frequently clustered rather than discrete, and the concept that distinct clinical phenotypes may exist in BS has recently emerged. The aim of the present work was to identify and analyze the disease phenotypes in a monocentric historical cohort of patients with BS.

**Methods:**

A total of 202 patients with BS diagnosis followed up at the Behçet Clinic of the Azienda Ospedaliero-Universitaria Pisana were identified, and demographics, clinical, and therapeutic data were retrospectively collected. Pairwise correlation among variables was evaluated by means of Pearson or Spearman correlation coefficient. A multiple correspondence analysis was performed to investigate the possible phenotypes resulting from the different patterns of associations among the demographic and clinical variables.

**Results:**

Most of the patients were female (67%), Caucasian (92%), and HLA-B51 carriers (65.5%). Mean age at disease onset was 30.06 ± 11.39 years, and oral ulcers (OU) and genital ulcers (GU) were the most common manifestations (96% and 61%, respectively). According to bivariate correlation analysis, significant positive correlations were observed between skin lesions and both OU (*p* = 0.005) and arthritis (*p* = 0.014), as well as pathergy (*p* = 0.001), gastrointestinal (GI) symptoms (*p* = 0.001), and other involvement (fever and serositis) (*p* = 0.015). Neurological involvement was significantly and positively associated with ocular lesions (*p* = 0.0114), GI symptoms (*p* = 0.030), pathergy (rho = 0.147, p = 0.037) and vein thromboses (*p* = 0.037). Despite the high heterogeneity, four disease phenotypes emerged from the MCA: (A) male Caucasians with greater age at onset and at diagnosis than the median values, with OU and GU, skin lesions, erythema nodosum (EN), arthritis, and GI symptoms; (B) co-existence of benign subset and pathergy; (C) orchitis/epididymitis associated with neurological involvement and ocular lesions; and (D) GI symptoms plus endoscopic lesions, large vessel disease (both arterial and venous), and other involvement.

**Discussion:**

This study provides valuable insights into the possible BS clinical phenotypes, and the results partially agree with previous association studies on European and extra-European cohorts. Observational comparative studies are warranted to assess the response of tailored phenotype-based therapeutic approaches.

## Introduction

Behçet’s syndrome (BS) is a rare multi-system vasculitis with a relapsing–remitting course, usually beginning in young adulthood. Recurrent oral and genital ulcerations (OU and GU, respectively) are the clinical hallmarks of disease, along with posterior uveitis (1, 2). Other common manifestations are papulo-pustular skin lesions, large joints arthritis, and vascular, neuro-psychiatric, and gastrointestinal (GI) alterations (3–7). Such manifestations show variable prevalence according to gender, age, and ethnicity (4, 8). Moreover, disease severity and prognosis are also highly heterogeneous depending on how the single manifestations cluster together resulting in distinct clinical phenotypes. Indeed, it is discussed whether BS should be considered as a complex disorder of distinct clinical phenotypes rather than a single nosological entity (9–11). The existence of different disease phenotypes is currently accepted, and it is supported by the numerous immunopathogenic pathways underlying distinct clinical manifestations, the highly heterogeneous presentation among patients from different geographies, and the differing response of different clinical manifestations to one same drug (12–15).

In the framework of the clinical variability that BS may have, four main well-recognized clinical phenotypes have been first suggested: (1) muco-cutaneous phenotype; (2) papulopustular and articular phenotype; (3) vascular phenotype; and (4) ocular phenotype (10, 16–18). More recently, several cohort studies have analyzed other possible combinations of disease manifestations, but study designs are heterogeneous and results are conflicting (19–26). However, identifying and describing disease phenotypes could contribute to further explore the diverse immunopathogenesis of BS manifestations, thus promoting a wider understanding of disease and earlier diagnosis for patients with BS. Moreover, the definition of clinical outcomes and therapeutic response within each BS phenotype might lead to improved and tailored therapeutic strategies based on the disease phenotype rather than on the single disease manifestations (10, 19). Ultimately, deeper insights into disease phenotypes contribute to accomplish effective overall management and better quality of life for patients with BS (27, 28).

The aim of the present work was to identify disease phenotypes in a monocentric cohort of patients with BS evaluating the possible associations between clinical, epidemiological, and therapeutic variables.

## Materials and methods

### Patients and data collection

This study was conducted at the Rheumatology Unit of the Azienda Ospedaliero-Universitaria Pisana in Italy. In this historical cohort, study patients were selected if they fulfill the following criteria: (i) BS diagnosis according to the International Study Group (ISG) and/or the International Criteria for BS diagnosis (ICBS) (29, 30), and (ii) regular follow-up at our Behçet Clinic. A total of 202 patients were included for the analysis using the outpatient clinic database and the medical charts. Demographic information, previous organ involvements, and therapies at last evaluation were retrospectively reported. The presence of the major histocompatibility complex (MHC) locus—HLA-B51—was also reported, when available. Organ involvement was defined as the presence of suggestive clinical presentation confirmed by performing imaging tests and according to a specialistic evaluation. However, the presence of GI disturbances consistent with the disease allowed the definition of GI involvement even in the absence of typical endoscopic lesions, provided the agreement with the gastroenterologist specialist.

The disease subset was classified as either benign or severe according to the previous organ involvement. Specifically, the presence of ocular manifestations (OM), neuro-psychiatric symptoms, GI, and/or vascular disease classified the disease subset as severe. Conversely, the presence of skin and mucosal lesions, arthralgias/arthritis, erythema nodosum (EN), and pathergy defined a benign disease.

### Statistical analysis

Quantitative variables were expressed as mean and standard deviation, while categorical variables were expressed as numbers (*N*) and percentages. The distribution of variables according to gender and disease course was evaluated considering the chi-square test or Fisher’s exact test as appropriate for categorical variables and using independent-samples *t*-test for quantitative variables. Pairwise correlation among variables was evaluated by means of Pearson or Spearman correlation coefficient as appropriate according to the type of variable.

To investigate possible distinct phenotypes resulting from different patterns of associations between the variables, a multiple correspondence analysis (MCA) was used. MCA is an explorative multivariate statistical method of dimension reduction for multiple categorical variables. It allows revealing the underlying structures in complex datasets - such as patterns of correlation among variables or similarities among observations, and providing a geometric interpretation through a spatial map of the data’s significant dimensions, where proximities between points and other geometric features indicate the associations among these dimensions (31). Patterns resulting from MCA are generally interpreted considering the first two dimensions extracted from the analysis, which represent most of the data variability displayed on two axes (the horizontal axis for the first dimension and the vertical axis for the second one). The graph allows for a visual assessment of the proximities between variables along each dimension, revealing existing patterns among them. Contributions of the different variables to each dimension were also obtained in order to understand the most influential variables contributing to the identification of the dimensions.

The advantage of MCA is that there is no need to meet assumption requirements.

All analyses were performed using the R package.

## Results

Characteristics of patients according to gender and subset of disease are shown in **Tables 1** and **2**, respectively. The majority of patients were female and Caucasian. Mean age at disease onset was 30.06 ± 11.39 years, and mean age at the time of diagnosis was 37.39 ± 10.94 years. HLA-B51 was available for 119/202 patients, of whom 78 (65%) were HLA-B51+. OU and GU were the most common manifestations (96% and 61%, respectively), while venous thromboses and neurological and GI involvement were less frequent. Disease subset was benign for 94 (47%) patients and severe for 108 (53%) patients, according to their previous organ involvement. Colchicine was the most used medication (44%), and adalimumab was the most used biotechnological disease-modifying anti-rheumatic drug (bDMARD) (21%).

**Table 1:** Patients’ characteristics according to gender.

	Women (*N* = 136, 67%)	Men (*N* = 66, 33%)	Total	*N*	*p*-value
Age at onset	29.39 ± 11.15	29.39 ± 11.15	30.06 ± 11.39	192	0.243
Age at diagnosis	31.45 ± 11.86	40.46 ± 12.42	37.39 ± 10.94	198	0.006
Caucasian	128 (94.81%)	60 (90.91%)	188 (93.53%)	201	0.29
HLA-B51+	57 (67.07%)	21 (61.76%)	78 (65.55%)	119	0.583
OU	133 (97.79%)	61 (92.42%)	194 (96.04%)	202	0.066
GU	91 (66.91%)	33 (50.77%)	124 (61.69%)	201	0.028
EN	34 (25.19%)	13 (19.70%)	47 (23.38%)	201	0.388
Skin lesions	67 (49.26%)	31 (47.69%)	98 (48.76%)	201	0.835
Arthralgias	67 (49.63%)	27 (40.91%)	94 (46.77%)	201	0.245
Arthritis	46 (34.07%)	20 (30.30%)	66 (32.84%)	201	0.593
OM	53 (38.97%)	25 (38.46%)	78 (38.81%)	201	0.945
CNS involvement	10 (7.35%)	10 (15.15%)	20 (9.90%)	202	0.082
GI symptoms	20 (14.71%)	8 (12.12%)	28 (13.86%)	202	0.618
GI symptoms plus endoscopic lesions	8 (5.88%)	6 (9.09%)	14 (6.93%)	202	0.4
Pathergy	20 (14.93%)	6 (9.38%)	26 (13.13%)	198	0.297
SVT	11 (8.09%)	4 (6.06%)	15 (7.43%)	202	0.778
DVT	10 (7.35%)	3 (4.55%)	13 (6.44%)	202	0.553
LVI	2 (1.49%)	3 (4.76%)	5 (2.54%)	197	0.33
Psychiatric symptoms	0	3 (4.55%)	3 (1.49%)	202	0.034
Headache	40 (29.42%)	10 (15.38%)	50 (24.88%)	201	0.031
Orchitis/epididymitis	0	4 (6.06%)	4 (1.98%)	202	0.011
Fever	27 (14.81%)	7 (10.77%)	27 (13.50%)	200	0.512
Benign subset	66 (48.53%)	28 (42.42%)	94 (46.54%)	202	0.454
Adalimumab	23 (16.91%)	19 (28.79%)	42 (20.79%)	202	0.064
Azathioprine	25 (18.38%)	17 (25.76%)	42 (20.79%)	202	0.268
Colchicine	66 (48.53%)	22 (33.33%)	88 (43.56%)	202	0.049
Golimumab	8 (5.88%)	4 (6.06%)	12 (5.945)	202	0.96
Certolizumab	8 (5.88%)	0	8 (3.96%)	202	0.055
Apremilast	9 (6.62%)	0	9 (4.46%)	202	0.032
Infliximab	17 (12.50%)	16 (24.24%)	33 (16.34%)	202	0.034
Canakinumab	2 (1.47%)	0	2 (0.99%)	202	1
Methotrexate	5 (3.68%)	5 (7.58%)	10 (4.95%)	202	0.231
Cyclosporine	1 (0.74%)	2 (3.03%)	3 (1.49%)	202	0.249
Istekizumab	1 (0.74%)	0	1 (0.50)	202	1

OU, oral ulcers; GU, genital ulcers; EN, erythema nodosum; OM, ocular manifestations; CNS, central nervous system; GI, gastrointestinal; SVT, superficial vein thrombosis; DVT, deep vein thrombosis; LVI, large vessel involvement.

**Table 2:** Patients’ characteristics according to the subset of disease.

	Severe subset (*N* = 108, 53%)	Benign subset (*N* = 94, 47%)	Total	*N*	*p*-value
Female patients	70 (64.81%)	66 (70.21%)	136 (67.33)	202	0.415
Age at onset	29.72 ± 11.42	30.45 ± 11.42	30.06 ± 11.39	192	0.659
Age at diagnosis	37.61 ± 10.62	37.14 ± 11.35	37.39 ± 10.94	198	0.763
Caucasian	100 (93.46%)	88 (93.62%)	188 (93.53%)	201	0.964
HLA-B51+	43 (63.24%)	35 (68.63%)	78 (65.55%)	119	0.54
OU	101 (93.52%)	93 (98.94%)	194 (96.04%)	202	0.049
GU	58 (54.21%)	66 (70.21%)	124 (61.69%)	201	0.02
EN	26 (24.30%)	21 (22.34%)	47 (23.38%)	201	0.743
Skin lesions	43 (40.19%)	55 (58.51%)	98 (48.76%)	201	0.01
Arthralgias	47 (43.52%)	47 (50.54%)	94 (46.77%)	201	0.32
Arthritis	35 (32.71%)	31 (32.98%)	66 (32.84%)	201	0.968
OM	75 (69.44%)	3 (3.23%)	78 (38.81%)	201	<0.001
CNS involvement	20 (18.52%)	0	20 (9.90%)	202	<0.001
GI symptoms	14 (12.96%)	14 (14.89%)	28 (13.86%)	202	0.692
GI symptoms plus endoscopic lesions	14 (12.96%)	0	14 (6.93%)	202	<0.001
Pathergy	12 (11.54%)	14 (14.89%)	26 (13.13%)	198	0.485
SVT	15 (13.89%)	0	15 (7.43%)	202	<0.001
DVT	13 (12.04%)	0	13 (6.44%)	202	<0.001
LVI	4 (3.77%)	1 (1.10%)	5 (2.54%)	197	0.376
Psychiatric symptoms	3 (2.78%)	0	3 (1.49%)	202	0.25
Headache	33 (30.84%)	17 (18.09%)	50 (24.88%)	201	0.037
Orchitis/epididymitis	4 (3.70%)	0	4 (1.98%)	202	0.125
Fever	16 (14.81%)	11 (11.96%)	27 (13.50%)	200	0.555
Adalimumab	28 (25.93%)	14 (14.89%)	42 (20.79%)	202	0.054
Azathioprine	15 (13.89%)	27 (28.72%)	42 (20.79%)	202	0.01
Colchicine	32 (29.63%)	56 (59.57%)	88 (43.56%)	202	<0.001
Golimumab	8 (7.41%)	4 (4.26%)	12 (5.94%)	202	0.388
Certolizumab	5 (4.63%)	3 (3.19%)	8 (3.96%)	202	0.726
Apremilast	2 (1.85%)	7 (7.45%)	9 (4.46%)	202	0.085
Infliximab	26 (24.07%)	7 (7.45%)	33 (16.345)	202	0.002
Canakinumab	1 (0.93%)	1 (1.06%)	2 (0.99%)	202	1
Methotrexate	9 (8.33%)	1 (1.06%)	10 (4.95%)	202	0.021
Cyclosporine	3 (2.78%)	0	3 (1.49%)	202	0.25
Istekizumab	1 (0.93%)	0	1 (0.50%)	202	1

OU, oral ulcers; GU, genital ulcers; EN, erythema nodosum; OM, ocular manifestations; CNS, central nervous system; GI, gastrointestinal; SVT, superficial vein thrombosis; DVT, deep vein thrombosis; LVI, large vessel involvement.

**Figure 1:**
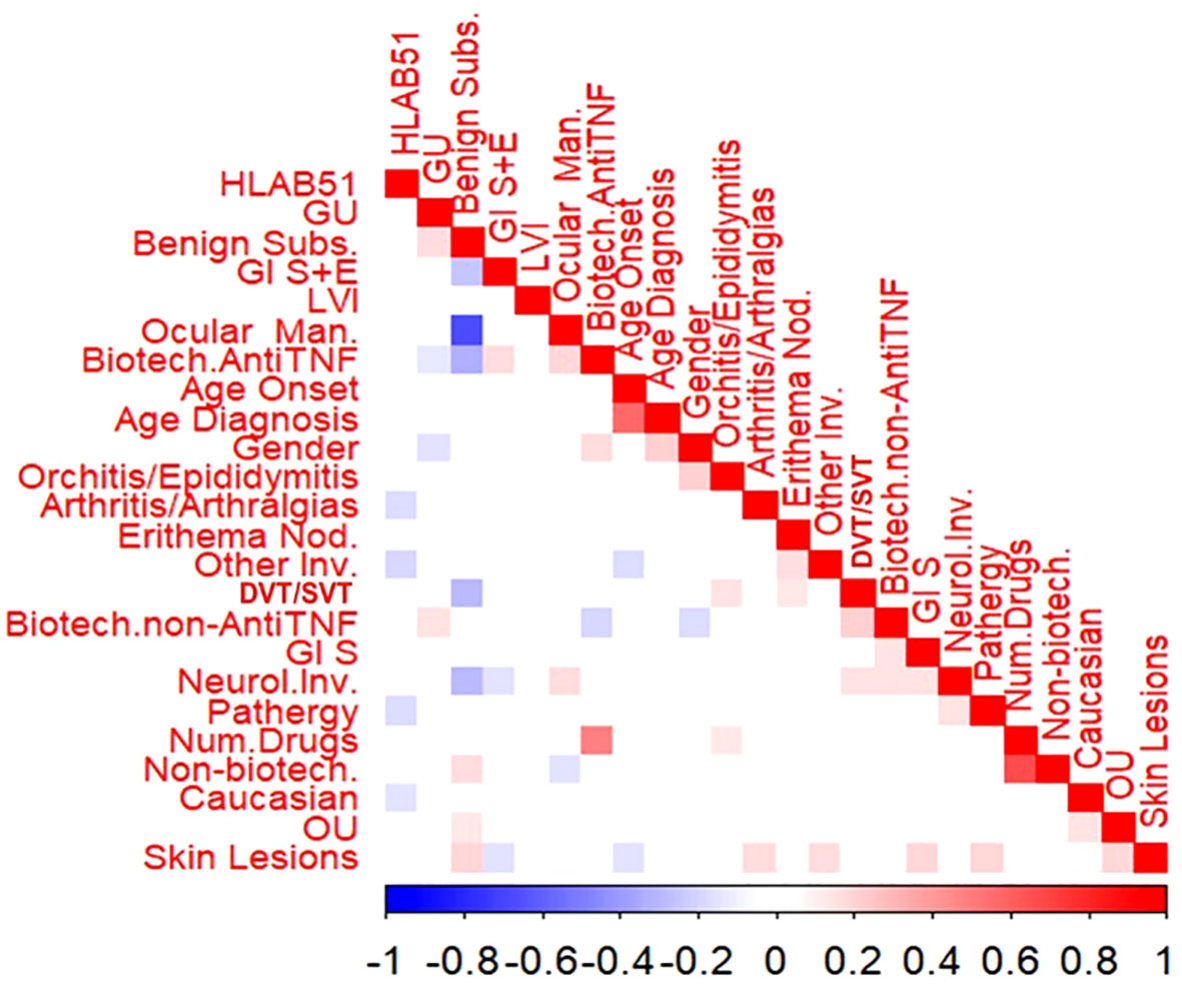
Bivariate correlations among the variables in the cohort. Male gender was significantly and positively associated with older age at diagnosis (rho=0.204, p=0.004), orchitis/epididymitis (rho=0.204, p=0.004) and the use of anti-tumour necrosis factor alpha (anti-TNFa) bDMARDs (rho=0.225, p =0.017), while the association was significantly negative with GU (rho=-0.149, p=0.034) and the use of non-anti-TNFa bDMARDs (rho=0.175, p=0.013). Caucasian race showed a significant negative association with HLA-B51 (rho=-0.146, p=0.033) and OU (rho=0.154, p=0.029). Age at onset and age at diagnosis were significantly and positively correlated (p=0.564, p<0.001); moreover, lower the age at onset higher the association with both skin lesions ( rho=- 0.158, p= 0.024) and other involvement (fever, costitutional symptoms and serositis) (rho=-0.160, p= 0.023). Benign subset was significantly and positively associated with GU (rho=0.160, p=0.023), OU (rho=0.139, p=0049) as well as skin lesions (rho=0.187, p=0.008). To what concerns organ involvement, a positive association was found between skin lesions and both OU (rho=0.197, p=0.005) and arthritis/arthralgia (rho=0.172, p=0.014), as well as pathergy (rho=0.189, p=0.001), Gl symptoms (rho=0.184, p-0.001) and other involvement (rho=0.187, p=0.015). Positive associations were also observed between thrombotic venous involvement and both erythema nodosum (EN) (rho=0.138, p= 0.049) and orchitis/epididymitis (rho=0.158, p=0.025). Neurological involvement was segnificantly and positively associated with OM (rho=0.172, p=0.0114), Gl symptoms (rho=0.153, p=0.0301), pathergy (rho=0.147, p= 0.037) and thrombotic venous involvement (rho=0.147, p=0.037). The association was significantly negative between Gl symptoms plus endoscopic lesions and both skin lesions (rho=0.148, p=0.036) and neurological involvement (rho=0.146, p=0.038) HLA-B51 revealed significant negative association with pathergy (rho=0163, p=0.045), arthritis/arthralgia (rho=-0.176, p=0.006), and other organ involvement (rho=-0.140, p=0.0.031).

The bivariate correlation analysis showed that few variables reported statistically significant correlations, as shown in **Figure 1**.

**Figure 2:**
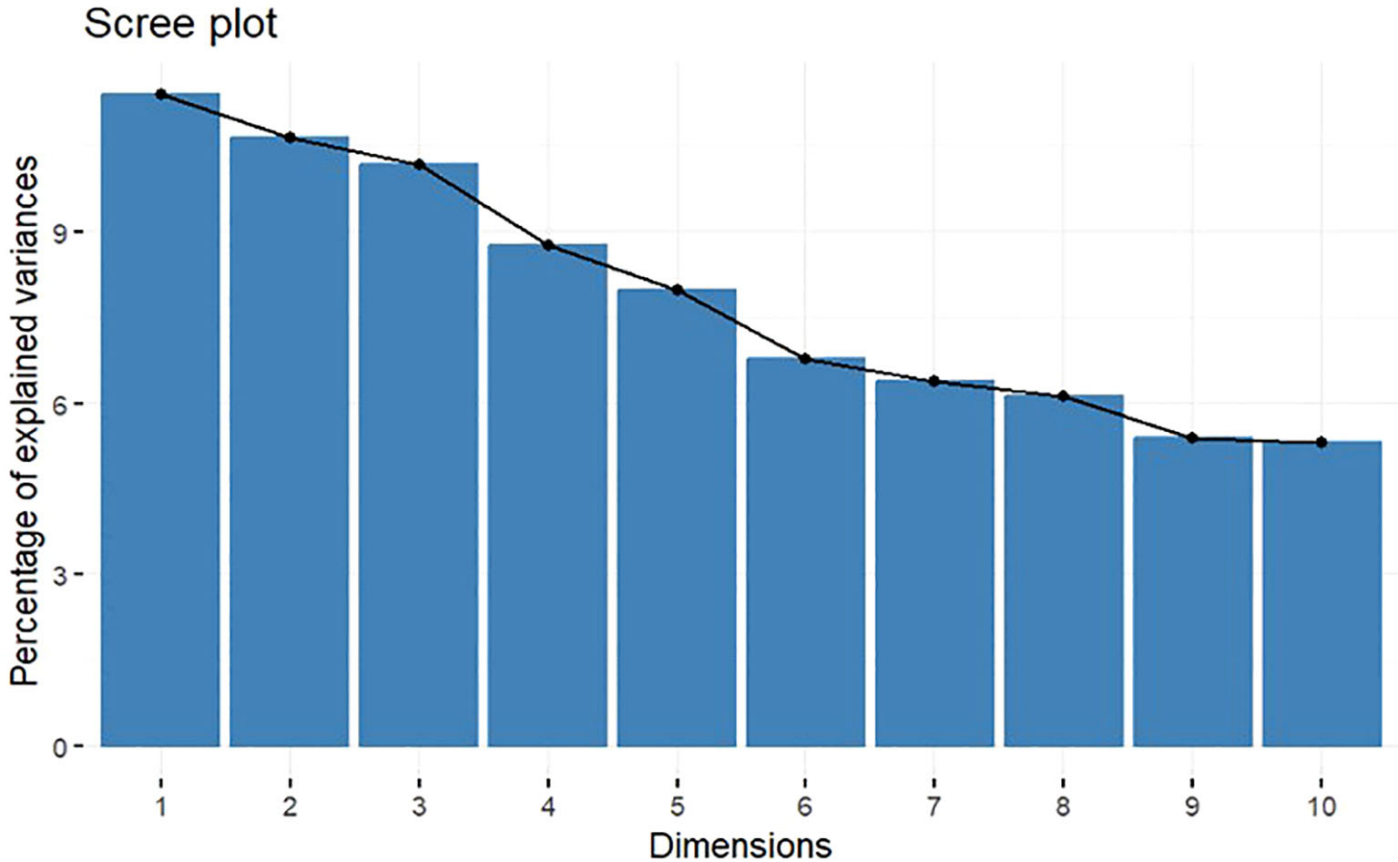
Screeplot. Percentages of explained variance for each dimension.

The MCA highlights the high degree of variability among observed data with 10 dimensions extracted explaining approximately 80% of the overall variability, with the first 2 dimensions capturing 22% of overall variability. Percentages of explained variance of the dimensions are shown in the scree plot in **Figure 2**. In particular, benign subset, skin lesions, OM, GI symptoms plus endoscopic lesions, pathergy, both arterial and venous disease [large vessel involvement (LVI)], deep and superficial vein thromboses, neurological involvement, and other involvement (such as fever, constitutional symptoms, and serositis) significantly contributed to the first two dimensions; thus, these variables mostly determined the different profiles that could be deduced from the analysis (**Table 3**).

**Table 3:** Contribution of the variables to the first two dimensions.

	Dimension 1	Dimension 2
Male gender	1.5	0.04
OU	0.16	0.06
GU	1.32	1.46
EN	0.14	1.17
**Skin lesions**	**5.41**	0.01
**OM**	**17.29**	**13.92**
GI symptoms	0.44	1.36
**GI symptoms plus endoscopic lesions**	**11.69**	**45.61**
**Pathergy**	1.02	**5.35**
**LVI**	4.03	**7.16**
Orchitis/epididymitis	2.87	2.93
**Other involvement**	0.69	**6.4**
**Benign subset**	**37.49**	1.82
Caucasian	0.02	0.25
Age at onset	0.01	1.67
Age at diagnosis	0.52	1.61
**DVT/SVT**	**11.58**	0.08
**Neurological involvement**	3.71	**8.22**
Arthritis/Arthralgias	0.12	0.87

OU, oral ulcers; GU, genital ulcers; EN, erythema nodosum; OM, ocular manifestations; GI, gastrointestinal; SVT, superficial vein thrombosis; DVT, deep vein thrombosis; LVI, large vessel involvement. The variables which significantly contributed to the first two dimensions are in bold.

**Figure 3:**
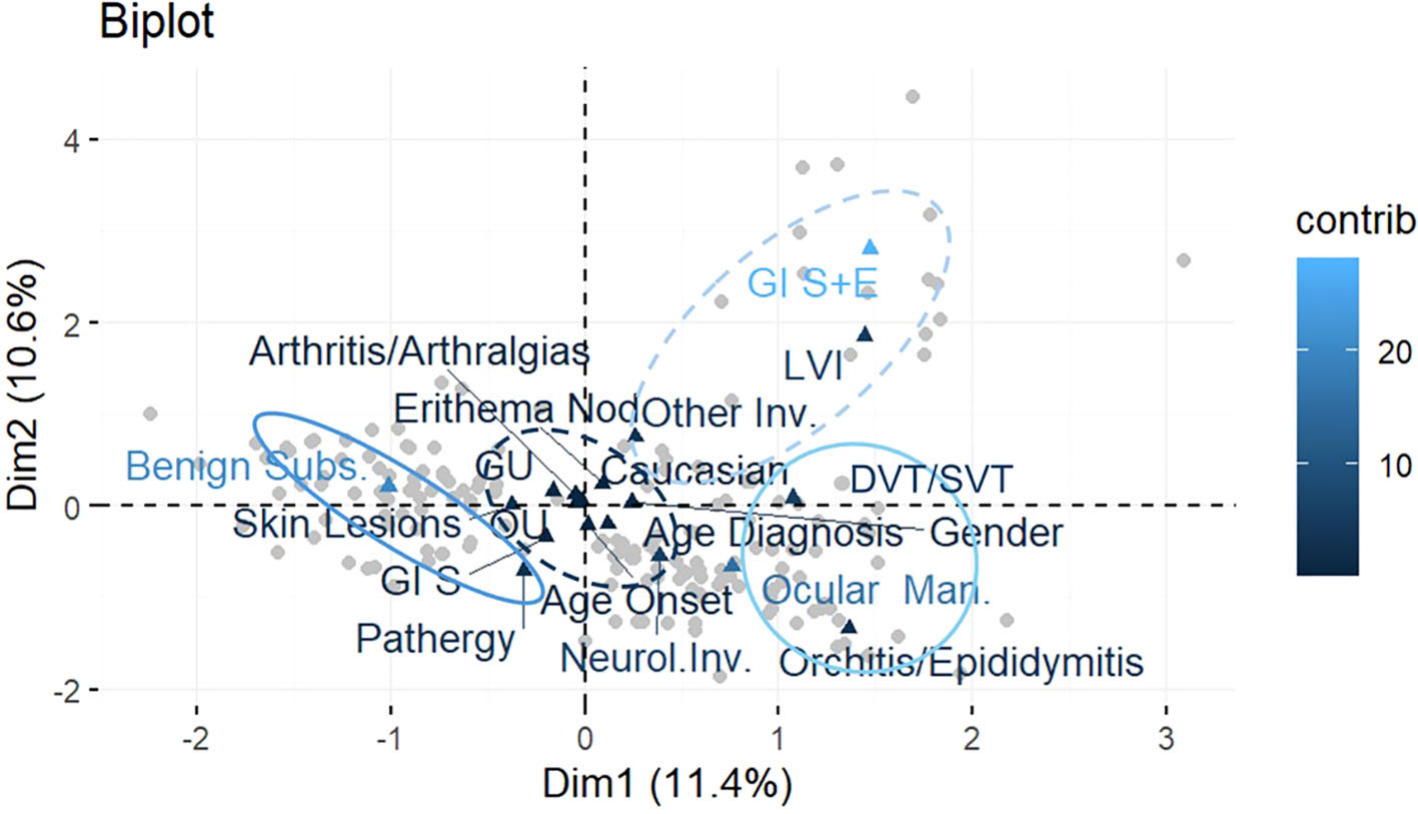
Biplot of observations and variables in the new system of coordinates identified with the MCA. Patterns of correlation between variables resulting from MCA are generally interpreted considering the first two dimensions extracted from the analysis, which represent most of the data variability. MCA allows to interpret the patterns of correlation between variables geometrically through a spatial map of the significant dimensions of the data displayed on two axes (the horizontal axis for the first dimension and the vertical axis for the second), where proximities between points and the other geometric features indicate associations between dimensions. Variables are highlighted in different colors according to their contribution to the two dimensions: the darkest the color, the highest the contribution.

According to the results, and applying the MCA, we were able to define four main distinct phenotypes in more detail (**Figure 3**):

- characterized by the presence of GI symptoms, skin lesions, OU and GU, arthritis/arthralgias, and EN, also being mainly male Caucasians, with age at onset and at diagnosis greater than the median values;
- characterized by the co-presence of benign subset and pathergy;
- characterized by the presence of orchitis/epididymitis associated with neurological involvement and OM; and
- characterized by GI symptoms plus endoscopic lesions, LVI, and other involvement (fever, constitutional symptoms or serositis).

## Discussion

The present work aimed at identifying and analyzing disease phenotypes in an Italian historical cohort of patients with BS. In agreement with other large BS cohort studies, patients included in the analysis were mostly women, the disease mainly occurred during early adulthood, and the muco-cutaneous involvement was the most prevalent (32–37).

Although there is large evidence from BS cohorts showing that severe manifestations more frequently occur in male patients (33–36, 38–40), we only observed a lower frequency of GU in male patients compared to female patients. Noteworthy, upon comparing this result with previous research involving European BS cohorts, we found that higher rates of uveitis, skin lesions, and venous thrombosis were observed in male patients (34, 35, 39) and that EN and arthritis were more common in female patients (34), with no significant difference in GU in both genders. However, gender did not significantly correlate with the benign or severe subset of disease in our analysis.

Moreover, older age at disease onset significantly correlated with skin lesions and other involvement, in contrast to previous studies that showed that patients with late disease onset frequently develop vascular, neurologic, and GI involvements compared to patients with early onset (41, 42). Last, we observed a negative correlation between HLA-B51 and pathergy, arthritis, and other involvement, while a meta-analysis of observational studies by Maldini et al. showed that HLA-B51 presence is mainly associated with decreased prevalence of GI involvement. Nevertheless, it is recognized that the contribution of HLA-B51 to the clinical presentation greatly varies with patient ethnicity (43).

To the best of our knowledge, this is the first study to assess the disease phenotypes in a BS cohort applying MCA. In the past years, multiple clinical phenotypes were identified performing cluster analysis and other association studies (16, 17, 44–47), but it has been argued that these methodologies may not be reliable for detecting existing disease phenotypes (23).

In this study, we were able to recognize four disease phenotypes. Specifically, phenotype A features a complete muco-cutaneous involvement associated with articular and GI manifestations in older male patients. As mentioned earlier, the association between skin lesions, mucosal ulcerations, and arthritis was widely described, as it is one of the first and most accepted disease phenotypes (17, 48, 49). Despite GI manifestations being conventionally severe, the absence of clear endoscopic findings suggests that this phenotype should be considered benign overall. Indeed, in clinical practice, it is not uncommon for patients with BS to refer non-specific mild abdominal symptoms that can be consistent with the disease. Phenotype B includes all the benign involvements of the disease. In contrast, patients of phenotype C show eye and neurologic involvement, in association with a less frequent manifestation like orchitis/epididymitis. Accordingly, Bitik et al. have previously reported a strong association between posterior uveitis and neurologic involvement (26), and other authors have recently observed a male predominance in ocular disease secondary to BS after performing a cluster analysis (44, 45).

Similarly, phenotype D is severe due to the presence of “complete” GI involvement (both symptoms and consistent endoscopic findings) and LVI, which consists in both venous and arterial lesions. Because of the very low prevalence of GI involvement, it was studied in few previous cluster analyses, and they showed that it rarely clusters with other organ involvement (45, 47). For what concerns LVI, strong association among peripheral venous and arterial lesions was found in previous research (50), as well as among peripheral vascular disease and cerebral venous sinus thrombosis (CVST) (51), leading to the definition of what is referred to as vascular or cardiovascular phenotype (3, 19). In addition, the vascular phenotype was mainly viewed as a separate phenotype in cluster studies (44, 47), and few associations with other clinical features were reported, such as a negative association with severe ocular lesions (20, 52). Of note, in phenotype D, vascular and GI involvement co-existed, in agreement with few other studies so far (53). Additionally, these two involvements suggest a highly inflammatory status; thus, unsurprisingly, auto-inflammatory symptoms like fever and serositis (referred to as other involvement in the current analysis) are also present in phenotype D.

This study provides valuable insights into specific BS clinical phenotypes, contributing to a more comprehensive understanding of BS heterogeneous clinical presentation. However, our study has some limitations, primarily due to the retrospective nature of the data. In addition, the monocentric design of our study might also have introduced potential selection biases. Another limitation is represented by the fact that these phenotypes are the result of artificial subgroups generated by the accumulation of clinical manifestations during the follow-up. Finally, considering that no significant associations were found in this regard, this type of analysis did not include ongoing therapies.

In conclusion, our analysis recognized four BS phenotypes within an Italian tertiary center. Our results add to prior evidence supporting the clinical perception that BS is a complex syndrome with different phenotypes consisting of different combinations of organ involvement. Phenotypes may have different pathogenetic mechanisms and different therapeutic implications. However, to date, pathogenetic studies in separate phenotypes of BS are still lacking, and comparative studies are needed to assess the response of tailored phenotype-based therapeutic approaches.

## Data Availability

The raw data supporting the conclusions of this article will be made available by the authors, without undue reservation upon reasonable request.
